# Is ergothioneine a ‘longevity vitamin’ limited in the American diet?

**DOI:** 10.1017/jns.2020.44

**Published:** 2020-11-11

**Authors:** Robert B. Beelman, Michael D. Kalaras, Allen T. Phillips, John P. Richie

**Affiliations:** 1 Department of Food Science, College of Agricultural Sciences, Center for Plant and Mushroom Foods for Health, Penn State University, 202 Rodney A. Erickson Food Science Building, University Park, PA 16802, USA; 2 Department of Biochemistry and Molecular Biology, Eberly College of Science, Center for Plant and Mushroom Foods for Health, Penn State University, 203A South Frear Building, University Park, PA 16802, USA; 3 Department Public Health Science, College of Medicine, Center for Plant and Mushroom Foods for Health, Penn State University, 500 University Dr., Hershey, PA 17033, USA

**Keywords:** Ergothioneine, Longevity vitamin, Fungi, Regenerative agriculture, Antioxidant, ERGO, Ergothioneine, ETT, ERGO transporter

## Abstract

There is mounting evidence for the potential for the natural dietary antioxidant and anti-inflammatory amino acid l-Ergothioneine (ERGO) to prevent or mitigate chronic diseases of aging. This has led to the suggestion that it could be considered a ‘longevity vitamin.’ ERGO is produced in nature only by certain fungi and a few other microbes. Mushrooms are, by far, the leading dietary source of ERGO, but it is found in small amounts throughout the food chain, most likely due to soil-borne fungi passing it on to plants. Because some common agricultural practices can disrupt beneficial fungus–plant root relationships, ERGO levels in foods grown under those conditions could be compromised. Thus, research is needed to further analyse the role agricultural practices play in the availability of ERGO in the human diet and its potential to improve our long-term health.

## Introduction

**Fig. 1.**
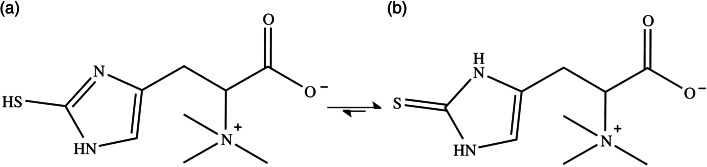
Structure of ergothioneine thiol (a) and thione (b) tautomers. At physiological pH, the thione form predominates.

Ergothioneine (ERGO) is a potent dietary antioxidant amino acid, first discovered in 1909 in Ergot fungus^(1)^. It has been shown to be an effective antioxidant partly because of the stability of its thione form which predominates at physiological pH^(2)^ (Fig. 1), making it resistant to autoxidation^(3,4)^. ERGO can scavenge reactive oxygen and nitrogen species and thereby mitigate oxidative damage to biological molecules that contribute to chronic human diseases^(5)^.

ERGO is currently gaining the attention of the scientific community as a result of its ability to reduce oxidative stress and act as an anti-inflammatory agent with the potential to serve as a therapeutic agent^(5)^. ERGO is not synthesised in animals or human subjects and is only obtained through dietary intake, leading us to consider it to be an important micronutrient. Limited intake of ERGO in the diet may compromise long-term health and life expectancy. In the present paper, we seek to illustrate the role ERGO may have in long-term human health and how limited dietary sources could be further impacted by agricultural practices.

## The role of ERGO in long-term human health

All mammals synthesise a cell membrane transport protein (in human subjects, the gene product of the SLC22A4 gene) which is highly specific for ERGO^(6)^. Prior to the discovery of the specificity for ERGO, the transporter originally was designated as OCTN1 or novel cation transporter, but it has repeatedly been shown to have a high affinity for ERGO and it more appropriately should be designated as the ERGO transporter (ETT)^(6–8)^. ETT is responsible for the rapid and efficient transport of food-derived ERGO from the intestine and into various tissues in the body. ETT is strongly expressed in erythrocyte progenitor cells in the bone marrow, small intestine, trachea, kidney, cerebellum, lung and monocytes^(7)^. In a bioavailability study, the uptake of ERGO into erythrocytes occurred within an hour of consumption^(9)^. ETT has been evolutionarily conserved through time leading to much speculation and interest about ERGO's importance to human health^(10)^.

Considerable attention has been focused on the potential for ERGO to mitigate chronic neurodegenerative diseases. One study demonstrated that ERGO blood levels in human subjects decline with age and declined faster in those who show cognitive impairment compared to age-matched individuals with no cognitive impairment^(11)^. In a similar study, blood ERGO levels were lower in individuals with Parkinson's Disease (PD) than with age-matched individuals without the disease^(12)^. These observations are not surprising considering that ETT is found in the brain and it has been demonstrated that ERGO passes the blood–brain barrier^(13)^, suggesting that low blood ERGO levels may predispose people to neurological diseases of aging.

**Fig. 2.**
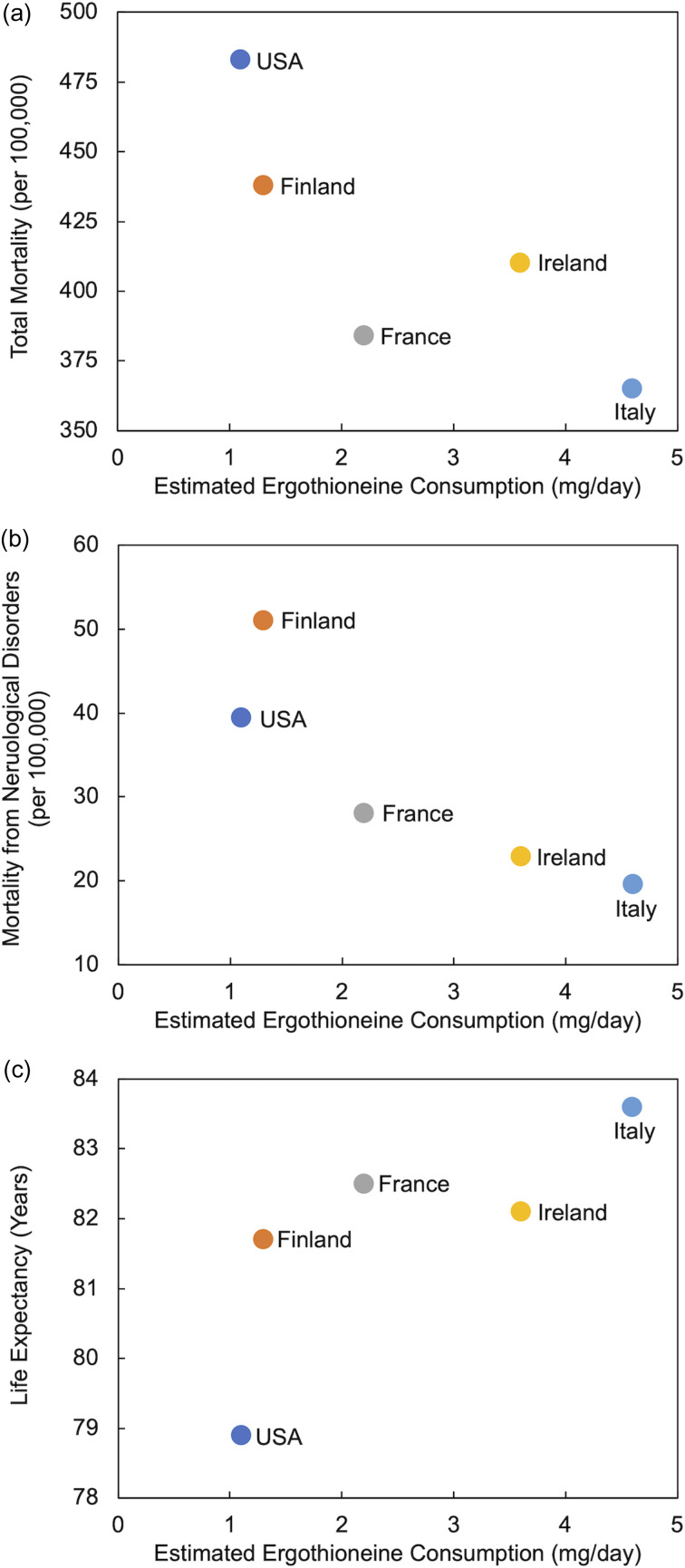
Estimated ergothioneine consumption in selected countries^(14,15)^ compared to (a) total annual mortality, (b) annual mortality from all neurological disorders (includes Alzheimer's disease and other dementias, Parkinson's disease, multiple sclerosis, epilepsy and other neurological conditions) and (c) life expectancy (data from WHO 2010)^(16)^.

Despite limited data on ERGO intake from the diet, Americans have been estimated to consume less ERGO (1⋅1 mg/day) than individuals in four European countries (up to 4⋅6 mg/day in Italy)^(14,15)^. These lower intakes were shown to coincide with a greater prevalence of chronic neurological diseases of aging and lower life expectancies^(15)^. In a similar analysis, utilising WHO data^(16)^ from 2010, we demonstrate in Fig. 2 that ERGO consumption appears to be negatively associated with total mortality and mortality from neurological disorders and positively associated with greater longevity. These associations cannot be interpreted as causal but have led us to the hypothesis that the American diet may be lacking in sufficient ERGO to adequately protect against chronic diseases of aging. Clearly, detailed ERGO intake data from other regions of the world are required to more fully examine these relationships.

Supporting our hypothesis, a recent longitudinal unbiased metabolomic study conducted in Sweden involving over 3200 adult men and women consuming a health-conscious food pattern at baseline sought to identify blood metabolites that could predict a lower risk of cardiovascular disease (CVD) and overall mortality^(17)^. Out of 112 metabolites measured at baseline, they found that plasma ERGO levels were the most strongly associated with decreased risk of CVD and reduced mortality after 21⋅4 years of follow-up. The authors proposed that raising plasma ERGO levels with diets containing more ERGO may reduce the risk of CVD and mortality. Another recent metabolomic study evaluated 131 blood metabolites as frailty markers in elderly hospital patients^(18)^. The metabolic profiles were clearly able to distinguish frailty from non-frailty and ERGO was lower in frail individuals than in those identified as non-frail. In addition, ERGO was also lower in those identified as having impaired cognition.

Numerous studies have demonstrated that mushroom consumption can be linked to decreased incidence of chronic diseases of aging such as dementia^(19,20)^, prostate cancer^(21)^ and risk factors of diabetes^(22–24)^. Given the strong associations observed between blood ERGO levels and mushroom consumption^(25)^, it is possible that many of these beneficial health effects of mushrooms could be attributed to ERGO. However, further research is required to support this speculation.

## ERGO as a vitamin

Dr. Bruce Ames recently suggested that ERGO should be considered a ‘longevity vitamin’ based on his Triage Theory that specifies that the human body uses certain micronutrients as though it were in a triage situation where priority is given to functions of reproduction and survival^(26)^. If an insufficient supply is available, then functions that support long-term health can be compromised, leading to reduced life expectancy. Such effects can be insidious, cumulative and not readily apparent but devasting just the same. In a similar light but a decade earlier, Paul and Snyder suggested that ERGO is ‘an important physiologic cytoprotectant which probably merits designation as a vitamin’^(27)^. However, ERGO does not meet the classic definition of a vitamin such that an inadequacy of it in the diet leads to an overt form of a disease in a short time frame. Contrary to the classic definition of a vitamin, researchers recently submitted the idea that since ERGO is commonly found in small amounts in nearly all foods but may not be present in sufficient amounts for optimum long-term health, it possibly could be considered as a vitamin^(28)^. We believe that ‘longevity vitamin’ is a more appropriate term for describing the role ERGO may play in human health.

## Sources of ERGO in the diet

Only certain fungi and a few other microbes^(28)^ are currently known to be able to produce ERGO. Since mushrooms are the fruiting bodies of fungi, it is not surprising that edible mushrooms are, by far, the leading dietary source of ERGO^(29)^. However, ERGO is also widespread in our food supply^(5,30)^ albeit in smaller amounts in most foods other than mushrooms^(29)^.

**Fig. 3.**
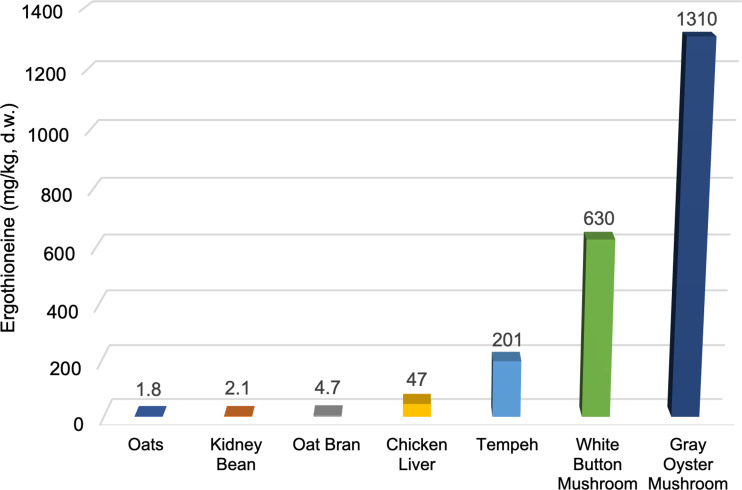
ERGO content of selected foods*.^(5,29,30)^. All values converted to mg/kg d.w. (dry weight).

Fig. 3 illustrates these large differences in ERGO content between mushrooms and some of the other foods that have been shown to contain measurable amounts. While the values of nutritional components in foods can vary from study to study, we have chosen representative ERGO values from the available literature for these foods. The ERGO content of commercially grown edible mushrooms such as white button (*Agaricus bisporus*) and gray oyster (*Pleurotus ostreatus*) mushrooms are much higher (630 and 1310 mg/kg d.w., respectively)^(29)^ than foods, such as oats^(5)^, kidney beans^(5)^ or chicken livers^(30)^, which contain some of the next highest documented levels (1⋅8, 2⋅1 and 47 mg/kg d.w., respectively). Tempeh, which is a soyabean product fermented with fungi, has been shown to contain moderately high levels of ERGO^(5)^ (201 mg/kg d.w.) but it is rarely consumed in the American diet compared to the other foods illustrated. Relatively low mushroom consumption, coupled with the predominant consumption of mushroom species with lower ERGO levels, may be partially responsible for the relatively low ERGO levels available in the American diet. In the U.S., *Agaricus bisporus* is by far the most consumed species of mushroom, with around 97 % of all mushrooms produced belonging to that species, compared to other mushroom species (such as oyster mushrooms) which typically have a much higher ERGO content^(15)^.

## Role of agricultural practices

**Fig. 4.**
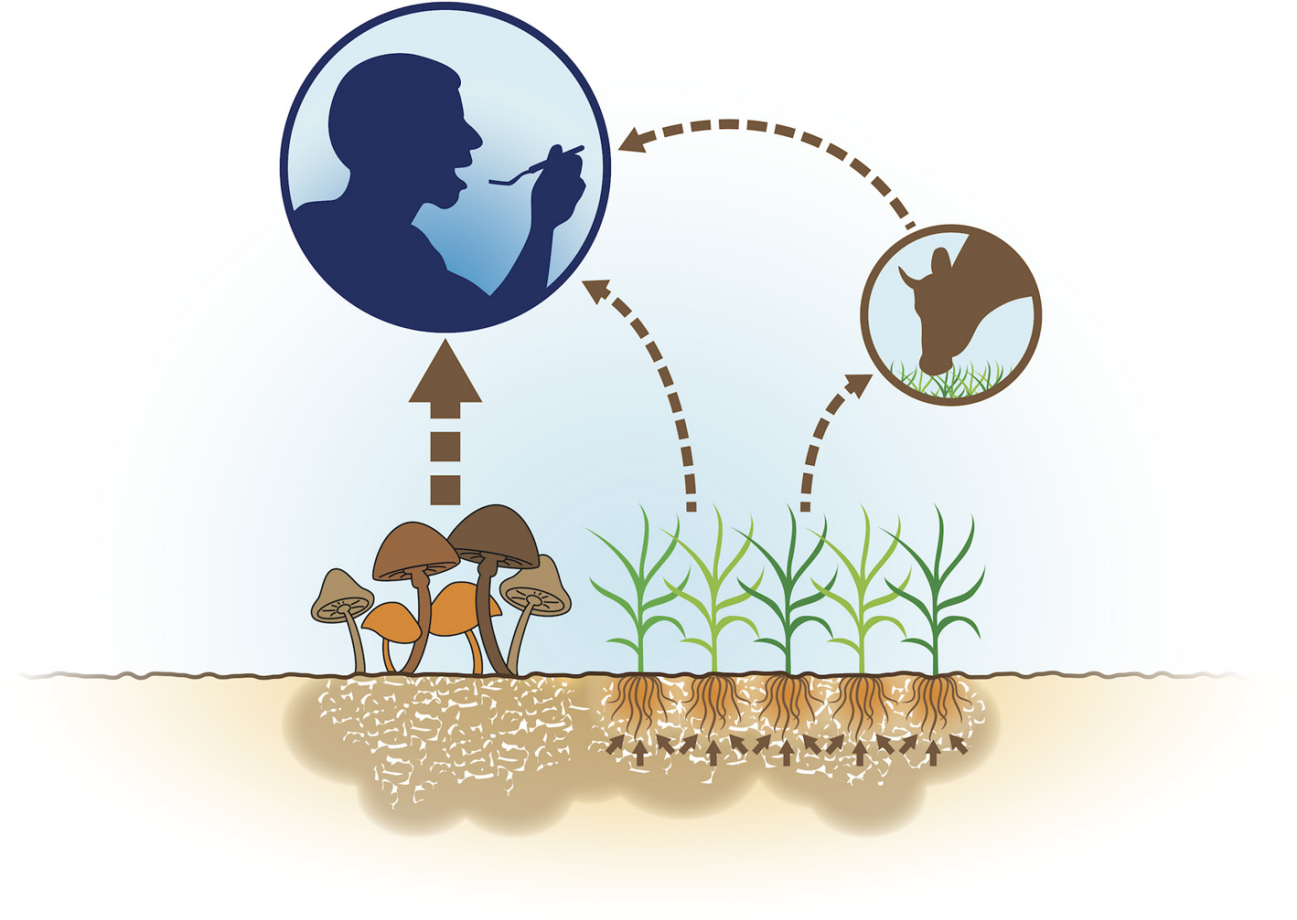
Sources of ergothioneine in the human diet originating from fungi in the soil.

It appears that some conventional agricultural practices may compromise the ERGO content of the diet. For example, excessive tillage of the soil, a common practice in conventional agriculture, may result in reduced ERGO content of crops. Excessive tillage is known to be disruptive to the mycelia of the mycorrhizal fungi in symbiotic association with the roots of crops grown in those soils^(31)^. Those mycelial networks are thought to be primarily responsible for passing ERGO on to plants as described previously^(32)^. A proposed schematic in which ERGO is distributed within our food supply through these interactions is demonstrated in Fig. 4.

Regenerative or restorative agriculture is a recent alternative to conventional agriculture that is considered more sustainable by employing no till, or minimal tillage, along with the use of cover crops, crop rotations and reduction in the use of chemical fertilizers and pesticides. Importantly, these practices have been shown to maintain healthy microbial populations in farm soils^(33)^. The regenerative agriculture movement has led to a burgeoning interest in evaluating the connection between soil health and human health. We believe that ERGO may be an important link since it is likely affected by agricultural practices.

## Conclusion

In conclusion, we believe that ERGO is a ‘longevity vitamin’ that is limited in the American diet, and likely other countries as well. This may be due to limited sources of ERGO in the diet and the implementation of agricultural practices that may have an impact on the ERGO content of the food supply. Hence, research is imminently needed to evaluate means to increase ERGO in the diet in order to improve our long-term health. One aspect of major importance is to determine if alternative agricultural approaches can successfully increase the ERGO content in the food supply.
